# An Automated Lexical Stress Classification Tool for Assessing Dysprosody in Childhood Apraxia of Speech

**DOI:** 10.3390/brainsci11111408

**Published:** 2021-10-25

**Authors:** Jacqueline McKechnie, Mostafa Shahin, Beena Ahmed, Patricia McCabe, Joanne Arciuli, Kirrie J. Ballard

**Affiliations:** 1 Faculty of Health Sciences, The University of Sydney, Camperdown, NSW 2006, Australia; tricia.mccabe@sydney.edu.au (P.M.); kirrie.ballard@sydney.edu.au (K.J.B.); 2 Faculty of Health, University of Canberra, Bruce, ACT 2617, Australia; 3 Faculty of Engineering, University of New South Wales, Sydney, NSW 2052, Australia; m.shahin@unsw.edu.au (M.S.); beena.ahmed@unsw.edu.au (B.A.); 4 College of Nursing and Health Sciences, Flinders University, Adelaide, SA 5042, Australia; joanne.arciuli@flinders.edu.au

**Keywords:** childhood apraxia of speech, motor speech disorder, prosody, lexical stress, automatic speech recognition, diagnosis

## Abstract

Childhood apraxia of speech (CAS) commonly affects the production of lexical stress contrast in polysyllabic words. Automated classification tools have the potential to increase reliability and efficiency in measuring lexical stress. Here, factors affecting the accuracy of a custom-built deep neural network (DNN)-based classification tool are evaluated. Sixteen children with typical development (TD) and 26 with CAS produced 50 polysyllabic words. Words with strong–weak (SW, e.g., **di**nosaur) or WS (e.g., ba**na**na) stress were fed to the classification tool, and the accuracy measured (a) against expert judgment, (b) for speaker group, and (c) with/without prior knowledge of phonemic errors in the sample. The influence of segmental features and participant factors on tool accuracy was analysed. Linear mixed modelling showed significant interaction between group and stress type, surviving adjustment for age and CAS severity. For TD, agreement for SW and WS words was >80%, but CAS speech was higher for SW (>80%) than WS (~60%). Prior knowledge of segmental errors conferred no clear advantage. Automatic lexical stress classification shows promise for identifying errors in children’s speech at diagnosis or with treatment-related change, but accuracy for WS words in apraxic speech needs improvement. Further training of algorithms using larger sets of labelled data containing impaired speech and WS words may increase accuracy.

## 1. Introduction

Difficulty with the production of lexical stress has been identified as one of the core deficits in both childhood and acquired apraxia of speech (CAS and AOS, respectively) [1,2] and has been studied for its potential as a diagnostic marker (e.g., [3,4,5]). Assessment of lexical stress production is traditionally impressionistic [6] and therefore vulnerable to various sources of error and bias within and between rater [7]. Objective acoustic measurement is advantageous for overcoming issues of perceptual bias or drift; however, manual measurement is time-consuming for clinicians [8]. This study aims to further the work of Shahin and colleagues [9,10,11,12] in the development of an automated lexical stress classification tool for CAS. Here, we compare a tool-based classification of stress patterns in isolated polysyllabic words (PSW) with expert auditory perceptual judgment given that lexical stress in PSW has proven sensitive to the differential diagnosis of CAS (e.g., [4]). We explore the potential for knowledge-driven systems to boost tool-based classification accuracy for mispronounced words and examine classification errors for potential segmental factors, which may affect tool accuracy and so guide stimulus selection for reliable assessment instruments in the future.

CAS is a speech sound disorder of neurological origin which affects the accuracy and consistency of the movements and movement transitions required for speech sound production [1]. The primary impairment is in the programming of the temporal and spatial parameters of movement sequences, manifesting in speech sound and/or prosodic errors [1]. Experts in CAS have reached some level of consensus around three segmental and suprasegmental features that are consistent with deficits in the programming of speech movements: “(a) inconsistent errors on consonants and vowels in repeated productions of syllables or words; (b) lengthened and disrupted coarticulatory transitions between sounds and syllables; and (c) inappropriate prosody, especially in the realisation of lexical or phrasal stress” [1]. 

Prosodic deficits continue to demonstrate significance as a valid diagnostic feature of CAS (e.g., [4,13,14]). Murray and colleagues [4] conducted a discriminant function analysis using a set of 24 quantitative measures extracted from a comprehensive clinical battery for diagnosing CAS. The gold standard comparison was expert diagnosis based on ASHA’s 3-item consensus-based feature list (described above) [1] and Strand’s 10-point checklist [15]. Perceptually judged error in producing lexical stress contrast in polysyllabic words was the strongest predictor of CAS diagnosis in the regression models presented [4]. This warrants development of an objective and efficient assessment tool for lexical stress to aid the clinical diagnosis of CAS. 

### 1.1. Lexical Stress

The English language uses lexical stress patterns in which stressed (strong) syllables and unstressed (weak) syllables tend to alternate both within words and across words within a phrase or sentence [16,17]. Over 90% of English words are polysyllabic (contain more than one syllable) (CELEX database, [18]) and therefore require control of stress contrast [19]. Most polysyllabic English words are classified as having either strong–weak stress (SW, e.g., DInosaur/ˈdaɪnəˌsɔ/) or weak–strong stress (WS, e.g., poTAto/pəˈteɪˌtoʊ/) over the first two syllables, with a tendency towards final syllable lengthening and medial syllable shortening [16]. Vowels in stressed syllables tend to be longer (msec) [16,17], louder (dB), and higher in fundamental frequency (f0) than vowels in unstressed syllables [20]. Duration and loudness make a greater contribution to listeners’ perception of prominence than fundamental frequency [20], especially in single-word picture-naming tasks [21]. Lexical stress in English can signal differences in grammatical function, such as noun (e.g., REcord) and verb (e.g., reCORD), and can be influential in spoken word recognition tasks (e.g., [22]). 

The majority of polysyllabic words in English carry initial stress (CELEX database; [18]). This statistical phenomenon most likely contributes to English-speaking children’s tendency to omit weak-onset syllables early in development [23] and the reduced intelligibility of apraxic speech where initial weak syllables are overstressed. 

Difficulty with production of lexical stress contrasts affects speech perception and speech intelligibility (e.g., [24]), reduces speech naturalness, and can lead to negative perceptions about the communicative competence of the speaker [25].

### 1.2. Measuring Lexical Stress 

Lexical stress is a good target for acoustic analysis as it involves manipulation of segmental or syllabic duration, fundamental frequency, and intensity—all variables that are readily measured by speech analysis software. Studies focused on acoustic analyses of lexical stress have also returned findings which support this as a key feature of CAS (e.g., [14,26,27,28]). While many of these studies did not directly compare acoustic and perceptual judgments of lexical stress, two reported no acoustic differences between typically developing and CAS groups in the production of lexical stress contrast, despite listeners perceiving less accurate stress production for speakers with CAS [26,27]. Skinder and colleagues [26] suggested that listener perception may have been influenced by segmental errors, while Munson and colleagues [27] proposed it may be influenced by the degree of difference in prominence across syllables. This proposal is supported by listeners’ tendency toward binary perception for stressed and unstressed vowels, despite acoustic measures showing a more graded scale, including de-stressed but nonreduced vowels [29]. 

Two acoustic measures of lexical stress have shown strong validity when directly compared with perceptual judgments of speech in CAS. Shriberg and colleagues [14] developed the lexical stress ratio (LSR, a single index generated from acoustic variables of vowel duration, intensity, and f0) and reported that inter-rater agreement for the global judgment of whether a child should be diagnosed as suspected CAS was higher when the child’s LSR fell in either the upper or lower extremes of the distribution [14]. Then, Ballard, Robin, McCabe, and McDonald [28] reported high agreement between perceptual judgment of lexical stress accuracy in words and their manually calculated normalised pairwise variability indices (PVIs; [30]). PVI is used to calculate the degree of asymmetry across two adjacent syllables in a string for vowel duration [30], as well as peak intensity or peak f0 [5,28,31]. It provides a measure normalised for individual articulation rate, vocal intensity, or f0, respectively. 

Advances in technology have made objective/acoustic analysis readily available through speech analysis freeware, such as Praat [32]. However, manual acoustic measurement is perceived to be too time-consuming for regular clinical use [8]. Many clinicians report that the analysis component of the assessment process is at least equally [33], if not more, time-consuming [34] than direct assessment activities. Therefore, development of an automated analysis system has high appeal. 

### 1.3. Automated Analysis of Lexical Stress

Automated acoustic analysis of word- and phrase-level lexical stress has been investigated for its potential to support both foreign language learning (e.g., [35,36]) and assessment and treatment of paediatric motor speech disorders [9,11,13,37], speech impairment [38], and autism [39]. Of those tools applied to disordered speech, studies have reported agreement with human judgment ranging between 10% and 77.6% ([38] and [11], respectively), or moderate to strong correlations [13,39]. We propose applying a threshold of 80% agreement between automated acoustic analysis and human judgment of lexical stress, as this is the threshold of clinically acceptable agreement often used between two human raters (e.g., [40]). Across both the language learning and speech disordered populations, the automated lexical stress analysis tools that achieved this 80% threshold have done so for correctly pronounced words (i.e., words with no segmental substitutions, distortions, deletions, or additions); however, these tools typically do not reach clinically acceptable standards when analysing mispronounced words (see [41] for a review). The best-performing tools reviewed by McKechnie and colleagues [41] that had been applied to mispronounced or disordered speech had generally used knowledge-driven methods, where the tools are supplied with data on the types of speech errors contained within the speech samples analysed. This type of specificity limits the wider clinical applicability of such tools and necessitates the use of confined dictionaries of words for analysis, as larger dictionaries will increase the phonetic neighbourhood of words and increase the likelihood of automated systems misrecognising a word based on phonetic similarity [42]. 

Shahin, Gutierrez-Osuna, and Ahmed [12] developed software which automatically classifies children’s lexical stress patterns across each adjacent syllable pair in isolated polysyllabic word productions. This tool calculates eight acoustic features for each syllable in a word, derived from the duration, f0, intensity, and spectral energy of two consecutive syllables:

1. Peak-to-peak Teager energy operator (TEO) amplitude over syllable nucleus;
2. mean TEO energy over syllable nucleus;
3. maximum TEO energy over syllable nucleus;
4. nucleus duration;
5. syllable duration;
6. maximum f0 over syllable nucleus;
7. mean f0 over syllable nucleus;
8. 27 Mel-scale energy bands over syllable nucleus.

These features are combined into a single wide feature vector and input into a deep neural network (DNN) classifier. From these combined features, the tool classifies each production as having either a SW, WS, SS, or WW stress pattern across adjacent syllables and assigns a confidence estimate for that classification, expressed as a proportion. The confidence estimate is a mathematical expression of the degree of certainty that a given word was produced with the recognised (i.e., automatically assigned) stress pattern. The tool does output pairwise comparisons across all syllables for a word, but consistent with work cited earlier, we focus here on the first two syllables. Typically developing children’s productions of three- and four-syllable polysyllabic words initiated with these four different stress patterns were entered into the DNN classifier with overall classification accuracy against dictionary-defined stress patterns reaching 88% [12]. Using a binary classification (SW, WS), the tool labelled stress patterns with 93% accuracy. However, for children with CAS, accuracy with the binary classification compared with human perceptual judgment was lower at 73.4%.

Shahin’s DNN tool [12] has advantages over previous models developed by the same team [10,11]. First, the tool was trained using child speech rather than adult speech. Second, the DNN classifier used raw syllable-level features rather than normalised PVI measures to learn more sophisticated relationships, and so reduces error rates compared with earlier versions [10]. 

Such automated tools have the potential to increase the objectivity, accuracy, and efficiency of speech analysis and clinical diagnosis. These findings offer support for the use of acoustic measures to profile prosodic difficulties and monitor treatment-related change. 

### 1.4. Purpose

This study is an extension of Shahin’s work [12], which compared classification accuracy for TD speakers with a dictionary-defined canonical stress pattern rather than with human judgment of the child’s actually produced stress pattern, and analysed only 15 words from 10 children with CAS. Here, we compare this same tool’s classification accuracy with human auditory perceptual judgment using speech samples from Australian English (AE)-speaking children with typical development and with CAS. We extend on earlier work by including a larger number of participants with CAS, and a wider range of three-, four-, and five-syllable polysyllabic words. We also perform deeper analysis of the tool’s classification accuracy using several methods. First, we explore the effects of pretraining the tool with information about specific phonemic/segmental errors made by the children, given the advantage for knowledge-driven methods identified in the review by McKechnie and colleagues [41]. There has been limited investigation of phonetic context as it applies to ASR using forced alignment. As such, we also explore the influence of phonetic contexts within words, given that syllabic nuclei are influenced by phonetic context and that phoneme boundaries may be more or less distinct depending on context [43]. Finally, we investigate the potential influence of the age of the speaker and the severity of speech impairment (as measured by percentage of phonemes produced correctly, PPC).

Our hypotheses were as follows:

1. An automated lexical stress classifier using acoustic features of duration, f0, intensity, and spectral energy across adjacent syllables in polysyllabic words will achieve:
  (a) ≥80% agreement with human perceptual judgments for TD speech;
  (b) Higher classification accuracy for TD speakers than for CAS speakers, for whom the likelihood of mispronunciation is high;
  (c) Higher classification accuracy when using a knowledge-driven system trained on the segmental errors represented in the disordered speech sample.
2. Classification errors will be associated with within-word features known to reduce human inter-rater reliability, such as equivocal stress across the first two syllables (e.g., HAMBURger/ˈhæmˈbɜgʌ/); short-vowel phonemes in the stressed syllable (e.g., BUTterfly/ˈbʌtəˌflaɪ); ambiguous phoneme boundaries (i.e., liquid consonants at syllable onsets or offsets such as in “elephant”); or words in which weak syllables have low intensity and/or undetectable pitch (i.e., unstressed vowels between two unvoiced phonemes, such as “potato”).

## 2. Materials and Methods

### 2.1. Participants

Sixteen typically developing children (7 males, 9 females; M = 6 years, range = 4–10 years, IQR = 3) and 26 children with CAS (22 males, 4 females; M = 4.5 years, range = 4–12 years, IQR = 3) participated. All children were Australian English speakers.

Typically developing children were recruited via convenience sampling from the local university community, and their inclusion criteria included: aged 4–12 years, parent report of typically developing receptive and expressive language skills, age-appropriate speech sound production skills as demonstrated by percent consonants correct (PCC; [44]) scores above 85% and developmentally appropriate phonology on the Single-Word Test of Polysyllables [45], no reported history of hearing deficits, no oromuscular structural deficits as indicated by age-appropriate oral structure and function scores on the Oral and Speech Motor Protocol [46], and no other developmental diagnoses.

Children with CAS were drawn from a cohort recruited for studies of CAS at a large metropolitan university. All children underwent a standard test battery for differential diagnosis of CAS [4]. This battery consisted of five commonly used and culturally appropriate (for Australian children) published tests: (1) the Diagnostic Evaluation of Articulation and Phonology (DEAP) Inconsistency subtest [47], used to assess token-to-token inconsistency in a single-word-naming task over 3 test administrations; (2) the Single-Word Test of Polysyllables [45,48], a 50-word picture-naming task used to assess speech sound accuracy, sound and syllable sequencing, and lexical stress accuracy; (3) a connected speech sample of at least 50 utterances, used to detect perceptual features of CAS in connected speech; (4) the Oral and Speech Motor Control Protocol including diadochokinesis (DDK; [46]); and (5) the Clinical Evaluation of Language Fundamentals (CELF, 4th edition, or Preschool, 2nd edition, Australian standardisations), used to assess receptive and expressive language skills [49,50]. Phonemic errors were determined using broad transcription and relational analysis (i.e., comparing the produced phonemes with the adult target production), taking age-appropriate developmental errors into consideration. Lexical stress errors were determined with reference to the operationalised definition provided by Iuzzini-Seigel, Hogan, Guarino, and Green [51]: “…the appropriate stress is not produced correctly…. if the stress is inappropriately equalized across syllables, or placed on the wrong syllable” (p. 40). Inclusion criteria included: aged 4–12 years; age-appropriate receptive language skills, indicated by a score of ≥85 on the receptive language index of the CELF-P2 [50] or CELF-4 [49]; no reported history of hearing deficits; no oromuscular structural deficits or evidence of dysarthria as indicated by age-appropriate oral structure and function scores on the Oral and Speech Motor Protocol [46]; and no other developmental diagnoses. Table 1 presents demographic information, speech production test data, and statistical comparisons for the participant groups. Note that the CAS group has significantly more males, reflecting the male bias for paediatric speech sound disorders [52]. 

### 2.2. Stimuli

Single-word stimuli included 50 colour pictures, each representing a common 3- to 5-syllable word ([45,48]; see Appendix A). Twenty-eight of the words are produced with unequivocal strong–weak (SW) stress across the first 2 syllables in Australian English (e.g., dinosaur, motorbike), 12 words with an unequivocal weak–strong (WS) stress pattern (e.g., tomato, banana), and 10 with a strong–strong (SS) stress pattern (e.g., hamburger, cucumber). The SS words typically involve some degree of stress contrast with primary and secondary strong stress but do not have the vowel reduction to schwa that is characteristic of weak syllables in Australian English. The Macquarie Dictionary Online for Australian English was used to categorise target stress patterns (www.macquariedictionary.com.au, accessed on 30 August 2017). The SS words were only included to analyse the tool’s confidence estimate of stress assignment, to provide a broader range of relative stress contrasts across syllables in the dataset. Words of 3 or more syllables were used in order to avoid conflating lexical stress pattern with final syllable lengthening effects in 2-syllable words [53].

### 2.3. Procedure

Each child was seated at a desk in a quiet room in the university speech pathology clinic or in their own home. Stimuli were presented via a PowerPoint presentation on a laptop computer. Slide advancement was controlled by the researcher, and for each slide, the child was prompted to name the picture. If the child did not produce the target word, he or she was first prompted with a forced-choice question (e.g., “Is it a watermelon or a pear?”) and, finally, with a cue for delayed repetition (e.g., “This is a watermelon. Now you say it”). This ensured a high response rate.

Speech samples were recorded with Audacity^®^ [54] or Praat [32] at 44,100 KHz sampling frequency using a Roland Quad-Capture UA-55 (Roland, Los Angeles, CA, USA) or Avid Recording Studio M-Audio Fast Track Audio Interface (Avid, Burlington, MA, USA) connected to a Dell Latitude laptop, and an adjustable head-worn microphone (AKG C520, AKG Acoustics, Vienna, Austria) at 5 cm mouth-to-microphone distance. Each word for each child was saved in a separate file, labelled with the target word (e.g., watermelon.wav), for batch processing with the lexical stress classification tool.

Prior to analysis, words that did not match the syllabic structure of the target word (e.g., productions with syllables deleted or added) were excluded. This was done for two main reasons: (1) the automated tool’s forced alignment procedure requires the correct number of syllables, and (2) the focus of this study was on lexical stress as defined by Iuzzini-Seigel and colleagues [51] and not on syllable production skills. For TD speakers, 0.86% of all sampled words were excluded at this step, and for CAS speakers, 22% of all sampled words were excluded (inter-rater reliability: 97% agreement; Cohen’s kappa = 0.98). Forty-five of the 50 individual stimulus words (see Appendix A) were amongst the excluded tokens, with 9.4% of these tokens having SS stress, 56.1% SW, and 34.5% WS. The higher rate for SW words is consistent with a predominance of SW words in the stimulus set. 

**Figure 1:**
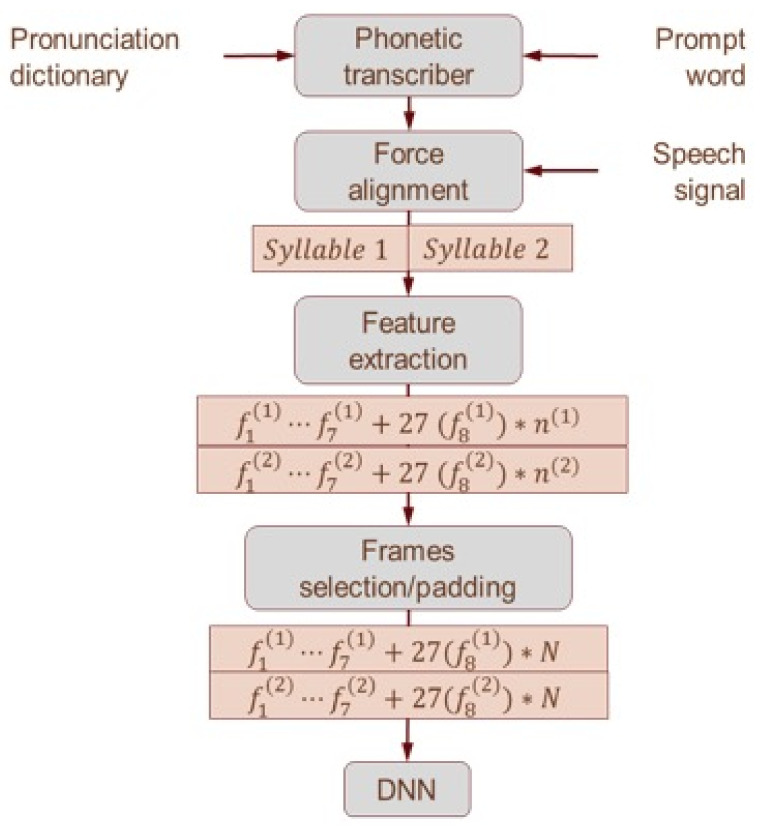
Flowchart depicting the automated classification process. *f_i_*^(1)^ and *f_i_*^(2)^ are the *i*th feature of the first and second syllable, respectively; n^((1)) and n^((2)) are the number of frames of the first and second syllables’ nuclei, respectively; and *N* is the number of input frames.

The set of productions that matched the target word syllable structure was run through the automated lexical stress classification tool. The tool consists of four main processes, which are described below and represented in Figure 1 (see also Table 2 below). 

#### 2.3.1. Forced Alignment

The tool took each individual wav file, linked to text information about that target word, and aligned it with the expected phoneme sequence. This sequence was extracted using a phonetic dictionary to estimate and mark phoneme boundaries within the word using the Gaussian Mixture Hidden Markov Model (GMM-HMM), an acoustic model, pretrained using the ANDOSL corpus of Australian English speakers [55]. The output of the forced alignment process is an estimated time boundary of each phoneme as well as nucleus and syllable durations.

#### 2.3.2. Feature Extraction

From each syllable, a set of eight acoustic measures known to correlate with lexical stress are extracted (see Table 2). Three energy-based features (*f*_1,_ *f*_2,_ *f_3_*) are extracted after applying the nonlinear Teager energy operator (TEO), which provides a better estimate of the speech signal energy and also reduces noise [56]. The pitch values are estimated using the autocorrelation method and the mean and maximum values computed over the duration of the nuclei [57]. These 7 stress detection features [58,59,60,61] are computed for each syllable, resulting in 2 values per bisyllabic pair. In addition, we computed Mel scale energies for each frame of the nucleus. 

#### 2.3.3. Concatenate Raw Features into 1 Wide Feature Vector

The tool then combined 8 acoustic features into 1 wide feature vector. Each syllable has 7 scalar values *f*_1_ − *f*_2_ and 27 × *n* Mel-coefficients, where *n* is the number of frames in each syllable’s vowel. To handle variable vowel lengths, we limit the number of input frames provided to the DNN to a maximum *N* frames for each syllable. This provides the DNN with a fixed-length Mel-energy input vector and allows the DNN to use information about the distribution of the Mel-energy bands over the vowel. If the vowel length (*n*) is greater than *N* frames, only the middle N frames are used. If the length of the vowel (*n*) is smaller than *N* frames, input frames are padded to *N* frames. The final size of the input vector to the DNN is 2 × (7 + 27 × *N*) for a pair of consecutive syllables, with *N* tuned empirically. 

#### 2.3.4. DNN Classifier

The vector for each word was input to the DNN classifier, which then categorised each word as either SW or WS, with an associated confidence level expressed as a probability. The DNN classifier was trained using the minibatch stochastic gradient decent method (MSGD) with an adaptive learning rate with data from the OGI corpus of American English children [62]. 

All samples were run through the classification tool twice: (1) the GMM-HMM model aligned the produced phoneme sequence against the expected sequence using a phonetic dictionary, which contained a *single* canonical representation of the target word (i.e., single-pronunciation HMM-based forced alignment), and (2) the GMM-HMM model aligned the produced phoneme sequence against the expected sequence using a phonetic dictionary, which contained *multiple* phonemic representations of the target words based on the range of actual variations/mispronunciations produced by the participants in the study (i.e., multiple-pronunciation HMM-based forced alignment). This was done on the hypothesis that mispronounced words may have generated errors in the forced alignment stage of processing, which, in turn, may have affected the feature vector analysis and subsequent stress pattern classification. Both sets of results were retained for analysis.

All productions were randomly ordered and played back to an experienced speech-language pathologist (the first author) for perceptual rating of stress pattern using a 5-point Likert scale (i.e., 1 = unambiguously weak–strong, 2 = somewhat weak–strong, 3 = equal stress, 4 = somewhat strong–weak, and 5 = unambiguously strong–weak). Following this, 48% of productions were randomly selected for independent rating by a second experienced speech-language pathologist (the last author) to establish reliability. Both raters were blinded to the output of the automated analysis at the time of rating. Rater 2 was also blinded to the participant group. Inter-rater reliability analysis was performed using the weighted Cohen’s kappa statistic [63]. The resulting reliability estimate indicated substantial agreement, K = 0.695 [64]. Prior to data analysis, perceptual ratings of stress patterns were collapsed to a 3-point scale, where 1 and 2 were combined into a single category coded 1 for WS, and 4 and 5 were combined to a single category coded 2 for SW.

### 2.4. Statistical Analysis

First, the primary dependent measure was the agreement between the tool and the primary human rater for lexical stress pattern (SW, WS) assigned to a word, where 1 indicated a match between automated and manual classifications and 0 indicated a mismatch. Percent agreement and Cohen’s kappa statistic [63] were used to describe the strength of agreement between the tool and the human rater by group, lexical stress type, and HMM-based forced alignment method (single-pronunciation HMM model vs. multiple-pronunciation HMM model), with data pooled across the participants.

Second, linear mixed model analyses were undertaken to determine whether the percent agreement between human and tool was predicted by the fixed factors of group (TD, CAS), stress type (SW, WS), and/or model type (single- or multiple-pronunciation HMM model), with the participant entered as a repeated factor. This approach was used as it is robust to missing data; WS percent agreement values were missing for 1 TD and 3 CAS children due to them having fewer than 3 tokens to calculate percent agreement after excluding responses with weak syllable deletion. Age was considered as a covariate. Post hoc testing with Sidak adjustment was undertaken to explore significant effects, the alpha level set at 0.05. An additional linear mixed effects model was run for the CAS group only, exploring PPC (i.e., speech disorder severity) as a covariate. All other components of the model were unchanged.

Third, to further explore the performance of the classifier on CAS data only, Mann–Whitney U tests were used to determine whether the lexical stress classifier had a different level of agreement with perceptual judgment for words perceptually scored by raters as having correct or incorrect lexical stress realisation. Incorrect productions were those receiving a score of 3 (equal stress) on the 5-point Likert scale described above or those where the perceived relative stress across the 2 two syllables was in the opposite direction to the dictionary-defined target stress pattern. The alpha level was set at 0.01 to adjust for multiple comparisons. Effect sizes were calculated using Hedges’ *g*, where values around 0.2 indicate a small effect, values around 0.5 indicate a medium effect, and values above 0.8 indicate a large effect [65].

Fourth, a series of correlation analyses were run for the TD and CAS children separately. Point biserial correlation, using the nonparametric Spearman’s rho statistic, was used to explore whether classification accuracy was associated with the tool’s confidence estimate for the assigned classification, or with the presence/absence of segmental features that may contribute to lower lexical stress contrastiveness or less reliable detection of phoneme boundaries. These features included nasal or liquid phonemes adjacent to the vowel, nonschwa unstressed vowels, or unvoiced plosives adjacent to an unstressed vowel, which can lead to low vowel intensity. In addition, post hoc analyses were conducted to further explore potential sources of classification error. We investigated whether classification accuracy was associated with age or phonemic accuracy, as measured by percent consonants correct (PCC), percent vowels correct (PVC), or percent phonemes correct (PPC) [44]. For these latter analyses, Spearman rho was used for the TD children due to non-normally distributed data, and Pearson’s correlation coefficient for the CAS group.

## 3. Results

### 3.1. Agreement between Classifier and Human Judgment

**Figure 2:**
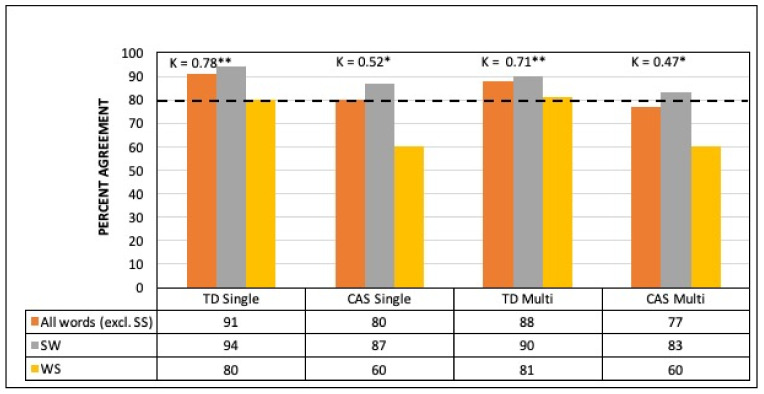
Percent agreement and Cohen’s kappa values for automated classification with single- vs. multiple-pronunciation HMM-based forced alignment compared with auditory perceptual judgment. TD = typically developing, CAS = childhood apraxia of speech, SS = strong–strong stress, SW = strong–weak stress, and WS = weak–strong stress, * = moderate effect, ** = substantial effect.

Figure 2 presents the percent agreement with human perceptual judgment for the automated lexical stress classification tool using single- and multiple-pronunciation HMM-based forced alignment in the TD and CAS groups. For TD children, the 80% agreement threshold was passed for both alignment methods for (i) pooled SW and WS words, (ii) SW words only, and (iii) WS words only. For CAS children, SW words reached >80% agreement under both alignment methods with WS words at about 60% agreement. Cohen’s kappa calculations on pooled SW and WS words showed substantial tool–human agreement for both single-pronunciation HMM-based forced alignment (κ = 0.78) and multiple-pronunciation HMM-based forced alignment (κ = 0.71). For the CAS children, agreement was moderate (κ = 0.52 and κ = 0.47, respectively).

### 3.2. Linear Mixed Effects Modelling

**Figure 3:**
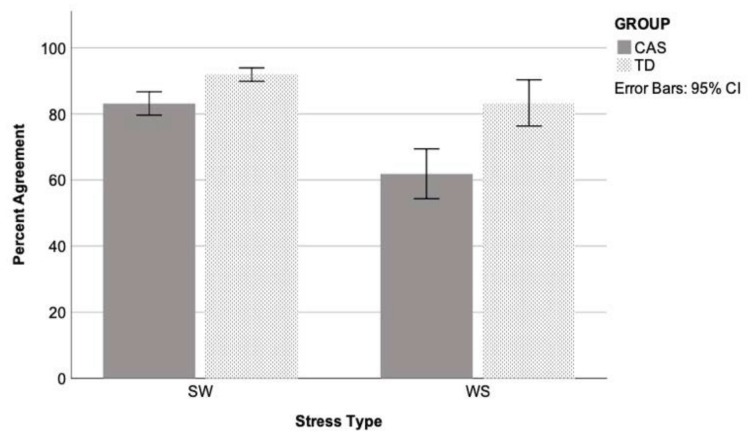
Percent agreement between human and tool for the typically developing (TD) and childhood apraxia of speech (CAS) groups across words with strong–weak (SW) and weak–strong (WS) lexical stress, pooled across single-pronunciation and multiple-pronunciation HMM model types. Error bars represent 95% confidence intervals.

Comparing across groups, an unstructured linear mixed model including the fixed effect of group (TD, CAS), the repeated effects of stress (SW, WS) and model (single, multiple), and the group by stress by model interactions, covarying for age (F(1,38.518 = 8.208, *p* = 0.007) was the model with the best fit, compared with a first-order regressive covariance structure with or without the covariate (see Table 3). Residuals were normally distributed. The main effects of group and stress type were significant, as well as the group by stress type interaction. Post hoc testing using the Sidak adjustment revealed that percent agreement was higher for the TD than CAS group for both stress types (see Figure 3), with the difference being greater for the WS words (mean difference for SW words = 8.471, *p* = 0.005, and for WS words = 22.937, *p* = 0.001). Within the TD group, percent agreement was not significantly different for SW and WS words (mean difference = 8.670, *p* = 0.114), but for the CAS group, agreement was higher for SW than WS words (mean difference = 23.137, *p* < 0.001). The stress by model interaction was close to significance (*p* = 0.053), likely due to a slightly higher agreement for the single- than multiple-pronunciation model for SW words only (SW mean difference = 4.588, *p* = 0.005, 89.98% and 85.39%, respectively; WS mean difference = 1.041, *p* = 0.688, 71.26% and 72.30%, respectively). 

To test for a possible effect of speech disorder severity in the CAS group only, a second model was run to analyse stress type and model type when PPC was entered as a covariate. The effect of PPC was significant (F(1,23.196) = 9.529, *p* = 0.005) (see Table 4). The effect of stress survived (see Figure 3), and model type continued to be nonsignificant (Model 1: SW = 86.15% average agreement, SE = 2.15, WS = 58.21%, SE = 5.71; Model 2: SW = 80.93%, SE = 2.33, WS = 61.14%, SE = 4.39). 

### 3.3. Words Perceived with Correct or Incorrect Lexical Stress

**Figure 4:**
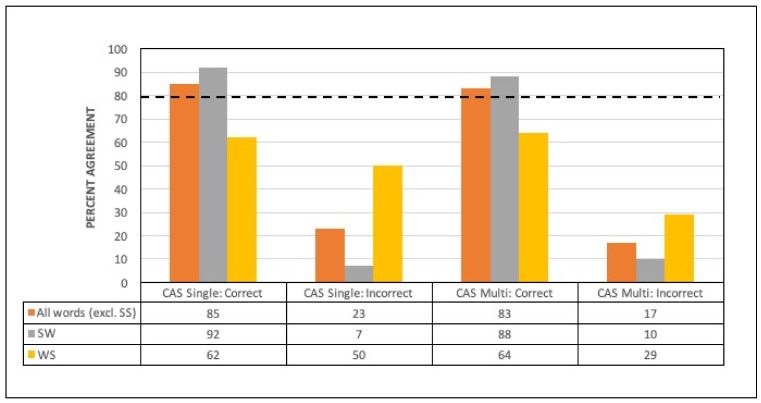
Percent agreement for automated classification with single- vs. multiple-pronunciation HMM-based forced alignment compared with auditory perceptual judgment, for words produced with correct and incorrect lexical stress. CAS = childhood apraxia of speech, TD = typically developing, SS = strong–strong stress, SW = strong–weak stress, and WS = weak–strong stress.

A total of 47 words from the analysed dataset were perceived as being produced with incorrect lexical stress (i.e., 18 WS and 29 SW words). These 47 words were produced by 18 of the 26 participants with CAS, with the median number of errors for the 18 participants being 3 (range, 1–5). Within the CAS group, SW words perceived by raters to have correct lexical stress realisation (i.e., rating of 4 and 5) showed high levels of agreement on stress classification with both the single- and multiple-pronunciation HMM-based forced alignment methods (92% and 88%, respectively; see Figure 4). For the SW words perceived as having equal stress (i.e., rating of 3) or incorrect/reversed assignment of lexical stress contrast, the classification agreement with the automated methods dropped significantly to just 7% and 10%, respectively (*p* < 0.0001; see Table 5). Although there was a tendency for higher tool–human agreement for WS words perceived as correct (i.e., rating of 1 and 2) versus incorrect for both forced alignment methods, this did not reach significance (see Table 5 and Figure 4). In all cases, agreement for WS words was well below the 80% threshold.

### 3.4. Confidence Estimates and Within-Word Features

Table 6 presents analyses of the relationship between tool–human agreement values (i.e., the tool’s classification accuracy), the tool’s confidence estimates of lexical stress classification for words, and within-word segmental features that were predicted to challenge the forced alignment procedure. For the TD samples, there was a strong positive correlation between classification accuracy and confidence estimate values using single-pronunciation HMM-based forced alignment and a weaker positive correlation for the multiple-pronunciation HMM-based forced alignment method. There was also a weak negative correlation between classification accuracy and presence of either a liquid or glide consonant adjacent to one of the vowels in a word (e.g., elephant) for the single-pronunciation method and a nonschwa vowel in the unstressed syllable (e.g., capsicum) for both the single- and multiple-pronunciation HMM-based forced alignment methods. The within-word features of long (e.g., motorbike) vs. short stressed vowel (e.g., butterfly) or unvoiced plosive (vs. voiced phoneme) plus schwa in the unstressed syllable (e.g., potato) were not associated with classification accuracy in this dataset.

For the CAS samples, there was a weak positive correlation between classification accuracy and confidence estimate using the single-pronunciation HMM-based forced alignment model and a moderate positive correlation for the multiple-pronunciation HMM-based forced alignment method. None of the correlations for within-word features reached significance (see Table 6).

### 3.5. Age and Severity

As the linear mixed model analyses revealed a significant effect of age and speech disorder severity on tool–human agreement, these variables were explored further. Table 6 also presents data on the associations between classification accuracy and the child’s age or speech disorder severity metrics. For the TD samples, there were no significant correlations between age and classification accuracy for SW or WS words for either single- or multiple-pronunciation HMM-based forced alignment. There were no significant correlations between PCC, PVC, or PPC and classification accuracy using single-pronunciation HMM-based forced alignment. Using multiple-pronunciation HMM-based forced alignment, there was a significant and moderate positive correlation between PCC and classification accuracy when SW and WS words were pooled.

For the CAS samples, classification accuracy was moderately correlated with age for WS words and for pooled SW and WS words using both single- and multiple-pronunciation HMM-based forced alignment. Classification accuracy demonstrated a moderate positive correlation with all three severity measures for both SW and WS words using the multiple-pronunciation HMM-based forced alignment model. Some comparisons were significant for the single-pronunciation method but with lower correlation values compared with the multiple-pronunciation method. 

## 4. Discussion

While there have been major advances in automated speech analysis within the past decade, this has focused almost exclusively on speech from healthy adults and on recognising words rather than identifying speech or prosodic errors. Automated acoustic analysis methods have great promise for improving the reliability of speech assessment for children with speech sound disorders and reducing the time burden of manual analyses for clinicians. Here, we compare the classification accuracy of the tool developed by Shahin et al. [12] with human auditory perceptual judgment using speech samples from a larger database of Australian English (AE)-speaking children with typical development and with CAS. We hypothesised that Shahin et al.’s automated lexical stress classifier would achieve ≥80% agreement with human perceptual judgments for speech of TD children but not for CAS and that a knowledge-driven system would outperform an unguided one. We also predicted that errors in automatic lexical stress classification would be associated with reduced stress contrastiveness and challenges to phoneme identification (e.g., liquid phonemes adjacent to vowels).

Our hypotheses were largely supported. The automated lexical stress classification tool achieved >80% agreement with expert auditory perceptual judgments for TD speech for both SW and WS stressed words. These results support previous studies exploring automated analysis methods with the speech of typically developing children (e.g., [10,36,58]). As predicted, the classifier demonstrated lower agreement with auditory perceptual judgments of lexical stress production in CAS speakers than TD speakers. These findings are consistent with Munson et al. [27] and Skinder et al. [26], who demonstrated a mismatch between acoustic evidence of lexical stress contrastivity and auditory-perceptual judgments of lexical stress accuracy in CAS. Given that tool–human agreement was associated with speech disorder severity, it is possible that segmental errors by the children with CAS may have affected the performance of the forced alignment in the automated procedure, or the perception of stress patterns by the human raters, as suggested by Skinder and colleagues [26]. Difficulty with control of relative timing of vowels in polysyllabic words in the children with CAS (e.g., [28,66]) may have contributed to these results, with the tool and possibly human raters being less reliable in assigning tokens to the SW or WS category as stress contrastiveness reduced. Human–tool agreement was particularly low for WS words produced by children with CAS. This is supported by Fear, Cutler, and Butterfield’s [29] findings that humans preferentially categorise de-stressed but unreduced vowels as strong vowels, possibly leading to less reliable categorisations of stress pattern. Computational neural modelling of speech motor control in CAS [67] demonstrated deviant coarticulation patterns in the speech of children with CAS, specifically longer and stronger carryover articulation from across adjacent phonemes. Such prolonged carryover coarticulation could have an impact on the accuracy of the forced alignment process of the tool and the subsequent extraction of features to determine lexical stress classification.

Here, classification accuracy for SW words from children with CAS also met the clinical threshold of >80% agreement with human raters, whereas previous findings from automated analyses of disordered speech samples have not met this clinically acceptable threshold [11,35,38]. However, classification accuracy for WS words from children with CAS was well below this 80% threshold. One possible reason is that producing segments of shorter duration is motorically more difficult than producing segments of longer duration (e.g., [21]). Acoustic studies on the development of lexical stress contrastivity show that typically developing children’s productions of WS words are not adultlike until the age of 12 years [68]. Children with CAS may make more significant phonemic mispronunciations as well as timing errors when attempting WS words, and these variations may contribute to poorer performance accuracy for the forced alignment processes of automated tools [41]. Such findings may explain the poorer performance of the tool for WS words in both TD and CAS groups in this study, although the classifier here was trained using child speech in the hope of mitigating the influence of maturation, and age was specifically included as a covariate in our analysis.

Programming the dictionary of the tool’s HMM-based forced alignment model with segmental information from the range of phoneme errors produced by the participants was trialled here to determine whether this improved the tool’s performance. There was evidence that the multiple-pronunciation model was influenced by the presence of speech sound errors, with a significant correlation between classification accuracy and disorder severity (i.e., PPC, PCC, and PVC) for the CAS group. When the variance due to disorder severity was controlled, there was no difference between the single- and multiple-pronunciation model’s performance. This suggests that a knowledge-driven approach may be useful in the further development of automated clinical speech assessment tools. Disorder severity aside, the single-pronunciation model outperformed the multiple-pronunciation model on measures of percent agreement with human judgment for both participant groups across most word categories. 

Our findings of improved classification accuracy for SW words produced with perceptually correct lexical stress patterns suggest that the version of the automated lexical stress classification tool tested in this study can determine stress patterns when productions are correct. While it was not able to reliably classify productions with reduced stress contrast across syllables, or equal stress, this may simply be a limitation of the tool’s design using a binary classification. However, these are the tokens that are of interest to clinicians, particularly given that children’s production of WS stress patterns continues to develop until age 12 [68]. It is possible that allowing classification to a third reduced or equal stress category would have improved the tool’s agreement with human raters. However, this was not true for WS words, where there was no significant difference in the tool’s performance between words produced with perceptually correct lexical stress and words produced with perceptually incorrect lexical stress. Further refinement of the classifier and testing on much larger samples of speech from typical and speech-impaired populations is required to increase its accuracy and diagnostic utility [69]. Future research may focus on determining specific cut-points along the continuum of stress contrast, where words with reduced stress contrast can be assigned to a third ambiguous (incorrect) classification. Here, we explored whether confidence estimates of the tool might guide the discovery of these cut-points, but this approach was not successful.

Although the spectral features extracted and filter banks used by the classifier were modelled on human speech perception and production, it is likely that there will continue to be differences between the human system and the modelled system for the foreseeable future. It is possible that there are differences between the acoustic features extracted by such algorithms and the features to which the human ear is attuned when judging lexical stress accuracy. For example, our study implemented a tool focused on proximal prosodic contrasts (i.e., relative differences across adjacent syllables), when it is likely that the human ear can attune to, and be influenced by, prosodic patterns across the entire speech stream (e.g., [70]), as well as the perceptual tendency for humans to make binary classifications of stressed versus not stressed for words in which the de-stressed syllable contains an unreduced vowel [29]. Another suggestion is that a computer-driven algorithm will seek to match the incoming signal to the pattern it has been trained to recognise, whereas human clinicians are trained to tune in to the incoming acoustic signal, regardless of expectation, and are able to use contextual information and sociological and linguistic factors to assist with parsing and perception of spoken language. One implication of these findings is that such tools may not yet be ready for integration into therapeutic applications until such time that they can provide accurate feedback on speech production, both correct and incorrect. 

Our findings for TD children indicate support for the hypothesis that classification errors are associated with more subtle lexical stress contrasts. Percent agreement with human judgment tended to be lower for words in which the unstressed vowel was not fully reduced to a schwa (i.e., when the word tended towards equivocal stress). While these syllables represent a separate and distinct acoustic category compared with stressed and unstressed syllables, the human ear has a tendency to categorise these with stressed syllables [29]. In contrast, classification accuracy was not significantly improved by removing words with equivocal stress from the CAS samples, nor was there any correlation between percent agreement and the within-word feature of a nonschwa unstressed vowel. This supports the hypothesis that children with CAS demonstrate reduced contrastiveness between syllables and tend towards equalised lexical stress [28]. It also lends support to the hypothesis that the perception of equal or excess stress in CAS may be a result of difficulty with control of relative timing as opposed to difficulty with the correct assignment of lexical stress (as suggested in [5,66]). 

For TD samples using the single-pronunciation model, classification error was weakly correlated with the within-word feature of liquid or glide phonemes adjacent to the vowel. This class of phonemes has the least distinct acoustic and spectrographic boundaries [5,71]. Here, coarticulation effects may result in inaccurate time boundaries during the forced alignment process, which will lead to inaccurate location of the vowel phoneme within the speech stream and extraction of acoustic features from the incorrect frame. This hypothesis was only weakly supported and did not hold true for both pronunciation models or in both participant groups. One possible solution would be to model the vowel and consonant together as one unit, in both the forced alignment process and the lexical stress classification model. This would require a redesign of the tool’s architecture.

Although age and severity were controlled for during linear mixed model analysis, their significant effects were explored more closely in the third set of analyses conducted here. Within-participant factors only partly explained our findings. Age was correlated with classification accuracy only for the CAS group, a finding likely to be due to the relationship between age and severity of speech impairment. Phonemic accuracy was moderately correlated with classification accuracy for some word types from the TD group using the multiple-pronunciation HMM model. As might be expected, phonemic accuracy was more influential in classification accuracy for the CAS group, where the likelihood of mispronunciation was high. Here, consonant, vowel, and overall phoneme accuracy each correlated moderately with tool classification accuracy in all word types for multiple-pronunciation HMM-based forced alignment with vowel accuracy also correlated with classification accuracy for all but the WS words in single-pronunciation HMM-based forced alignment. Using percent consonants correct as a measure of severity of speech involvement [44], classification accuracy was reduced as severity of speech impairment increased but only for multiple-pronunciation HMM-based forced alignment. Vowel accuracy was more significantly correlated with the tool’s performance accuracy across the range of tool and word types. This was to be expected given that the vowel is the nucleus of the syllable, and the tool performed its analysis of lexical stress at the syllable level. It was surprising that phonemic accuracy was more influential to performance accuracy of multiple-pronunciation HMM-based forced alignment than to the accuracy of single-pronunciation HMM-based forced alignment. Since the dictionary in this model of the tool had been primed with information about the phonemic variations produced by the participants, one would expect to have a reduced likelihood that mispronunciations would affect the tool’s ability to correctly classify lexical stress. Based on these data, this is not the case.

One possible reason that the multiple-pronunciation HMM-based forced alignment system did not significantly improve lexical stress classification accuracy is that the acoustic model was trained on adult speakers. This may have caused alignment problems if, instead of recognising mispronounced words, the aligner corrupted correctly produced words where the phoneme sequence was actually matched to a sequence in the single-pronunciation forced alignment system. Another explanation may be the fact that increasing the size of the dictionary resulted in higher error rates based on erroneous activation of phonetically similar targets [42]. However, it is likely that factors other than phonemic mispronunciation and lexical stress errors are influencing automated classification accuracy, as vowel and phoneme errors accounted for approximately 26% of the variance in classification accuracy in both the single- and multiple-pronunciation HMM-based forced alignment models. 

### Limitations and Future Directions

This research raises as many questions as it has answered. Further research should investigate whether children with CAS make more significant segmental errors and timing errors in their productions of WS words and the influence this has on automated lexical stress classification accuracy. Our dataset was unbalanced, with more SW words sampled than WS words. This was due to the facts that: (i) SW polysyllabic words are more common in English, particularly in nouns, while the WS pattern tends to be more common in verbs [72,73]; (ii) the children were sampled using a picture-naming task, which resulted in the dataset being comprised of nouns (i.e., picturable words) and therefore made up of more SW words than WS words; and (iii) stimuli in picture-naming tasks are limited to words that children will be familiar with. Future research could include a larger sample of WS words, particularly those produced with perceptually correct lexical stress to explore factors related to the tool’s significantly poorer performance on WS words. 

Further exploration of the similarities and differences between acoustic features extracted by machine learning algorithms and those to which the human ear is attuned when judging lexical stress accuracy is warranted. This would aid in determining why the algorithm does not match human perception, particularly for words with inaccurate stress patterns. 

Deeper analysis of the phonemic errors and their influence on syllable structure is required to further explain the finding that priming the acoustic model with specific knowledge about the types of mispronunciations in the speech samples offered only partial advantage to the tool’s classification accuracy.

The HMM-based forced alignment process of the tool was trained using adult speech samples so that the phoneme segmentation process was not affected by accent differences. This module of the tool may need to be further trained or adapted using data from children. Future directions for this research include directly testing the forced alignment component of the tool by comparing the sequence of recognised phonemes with the sequence of phonemes actually produced by the child. 

While the HMM-based forced alignment process was trained using Australian English speech, the DNN-based classifier was trained using a corpus of typically developing children using the US English dialect. This introduced the potential to negatively affect classification accuracy. While the influence of accent needs to be directly tested, US English and Australian English are dialectical variations of the same stress-timed language and therefore have similar alternating lexical stress across adjacent syllables. The model currently detects when a child clearly destresses a strong syllable or erroneously stresses a weak syllable. To enhance its accuracy in detecting more subtle lexical stress errors associated with the tendency towards excess and equal stress, the model needs to be trained using speech samples from children with CAS. As the amount of labelled data available for training increases, so too does the potential for improved accuracy of the algorithms.

There are some limitations inherent in using a forced alignment system. One is that phonemes undergo coarticulatory adjustments so that any given phoneme will vary based on its phonetic context. Therefore, phoneme boundaries are rarely discrete moments in time but estimates of best fit. This is particularly the case for phonemes such as liquids/glides transitioning into or out of vowel phonemes [71]. Another is that such systems require a constrained vocabulary and can only match the incoming speech signal to words within the predefined dictionary. Additionally, the system requires adequate training such that it can recognise words even when produced with speaker-dependent variations in the speech signal [71]. Constraining tasks and vocabulary to reduce the potential sources of variability in the speech signal may increase computerised analysis accuracy. However, it also has the effect of limiting the ecological validity of the speech sample and reducing the clinical utility and widespread application of computerised analysis processes if an ‘off the shelf’ tool cannot readily be applied to different populations and different word sets [71].

Further research could consider improving the acoustic model used in the forced alignment module of the tool. One way to achieve this would be to use a more advanced acoustic model based on deep learning [74]—or alternatively, to use domain adaptation techniques, suitable in instances where limited data from the target population are available, to adapt an acoustic model built on adult speech to children’s speech or disordered speech [75].

## 5. Conclusions

This study has the potential to guide the development of a test of lexical stress production for children, with an associated automated analysis tool for diagnosis relative to normative and other-disorder populations. Error analysis can provide guidelines for refining the tool to maximise sensitivity and specificity, for example, by examining classification errors for potential segmental factors, which may affect tool accuracy and so guide stimulus selection for reliable assessment instruments in the future. Such automated analysis tools may make the analysis of lexical stress difficulties more accessible to clinicians who have limited time and experience with acoustic analyses. This may be especially salient considering the availability of easily accessible technology to capture high-quality audio recordings within the clinic.

Automated speech analysis remains a difficult problem for clinical populations in the current state of technological development. However, the promising results from TD samples and CAS samples of SW words in the current study suggest that, once trained on larger datasets of disordered speech and with a greater range of WS exemplars, such tools have the potential to reach clinically acceptable benchmarks of accuracy against human raters in the near future.

## Figures and Tables

**Table 1:** Participant demographic and diagnostic speech production data.

Variable	TD (*n* = 16)		CAS (*n* = 26)		Statistics ^a^
	M (SD)	Range	M (SD)	Range	
**Demographic**					
Age (years)	6.1 (2.0)	4–10	5.9 (2.5)	4–12	*Z* = −0.71 ^ns^
Sex	7 male 9 female		22 male 4 female		*χ* = 7.7 *
**Test of Polysyllables ^b^**					
PPC	95.2 (4.2)	85.6–99.3	61.8 (21.1)	23.9–96.7	*t* = 6.24 **
PCC	95.4 (4.8)	81.4–100	57.5 (24.7)	13.0–98.6	*t* = 5.66 **
PVC	93.9 (5.3)	82.5–100	67.5 (17.6)	38.5–94.2	*t* = 5.82 **
% Lexical stress matches	88.8 (8.4)	77.3–100	51.0 (26.6)	6.3–93.8	*t* = 5.5 **
**Severity Rating ^c^**					
Typical–mild	15/16		5/26		
Mild–moderate	1 ^d^		5		
Moderate–severe	0		5		
Severe	0		11		

Note: ^a ns^ not significant at *p* < 0.05, * *p* < 0.01, ** *p* < 0.001; ^b^ Gozzard, Baker, and McCabe (2008), percent phonemes (PPC), consonants (PCC), or vowels (PVC) correct; ^c^ based on PCC from the Test of Polysyllables (>85% correct is typical–mildly impaired, 65–85% is mild–moderate, 50–65% is moderate–severe, <50% is severe); ^d^ participant sp011 was the youngest participant, aged 4 years—all errors were developmentally appropriate.

**Table 2:** The extracted acoustic features.

Feature	Description
*f*_1_	Peak-to-peak TEO amplitude over syllable nucleus
*f*_2_	Mean TEO energy over syllable nucleus
*f*_3_	Maximum TEO energy over syllable nucleus
*f*_4_	Nucleus duration
*f*_5_	Syllable duration
*f*_6_	Maximum pitch over syllable nucleus
*f*_7_	Mean pitch over syllable nucleus
*f*_8_	27 Mel-scale energy bands over syllable nucleus

Note: TEO = Teager energy operator.

**Table 3:** Test of fixed effects exploring the influence of group (TD, CAS), stress type (SW, WS), and model (single, multiple) on tool–human percent agreement for lexical stress classification, accounting for the effect of the child’s age.

Source	Numerator *df*	Denominator *df*	F	*p*
Intercept	1	49.346	365.151	<0.001
Group	1	32.858	18.645	<0.001
Stress	1	35.302	20.836	<0.001
Model	1	36.087	1.235	0.274
Age (covariate)	1	38.518	8.208	0.007
Group × Stress	1	35.328	4.314	0.045
Group × Model	1	36.084	0.118	0.733
Stress × Model	1	34.934	4.007	0.053
Group × Stress × Model	1	34.946	0.715	0.404

**Table 4:** For the group with childhood apraxia of speech (CAS) only, test of fixed effects exploring the influence of stress type (SW, WS), and model (single, multiple) on tool–human percent agreement for lexical stress classification, accounting for the effect of speech disorder severity (i.e., percent phonemes correct, PPC).

Source	Numerator *df*	Denominator *df*	F	*p*
Intercept	1	29.545	88.358	<0.001
Stress	1	20.894	22.864	<0.001
Model	1	22.420	0.214	0.648
PPC (covariate)	1	23.196	9.529	0.005
Stress × Model	1	20.592	3.362	0.081

**Table 5:** The lexical stress tool’s accuracy against human judgement for children with childhood apraxia of speech (CAS), for words perceived by raters as having correct versus incorrect stress production.

	Single Pronunciation			Multiple Pronunciation		
Comparison	Statistic	*p*	*g*	Statistic	*p*	*g*
All (excl. SS)	*U* = 26 *z* = 4.95	<0.0001	3.215	*U* = 25 *z* = 4.98	<0.0001	3.617
SW	*U* = 0 *z* = 5.48	<0.0001	7.468	*U* = 0 *z* = 5.48	<0.0001	6.683
WS	*U* = 131.5 *z* = 0	1	0.079	*U* = 78.5 *z* = 1.17	0.242	0.430

Note: SS = strong–strong stress pattern (e.g., hamburger), SW = strong–weak (e.g., dinosaur), WS = weak–strong (e.g., tomato), alpha was set at 0.01 to adjust for multiple comparisons.

**Table 6:** Correlation between classification accuracy for words using the single and multiple pronunciation HMM-based forced alignment methods and (a) the tool’s confidence estimates in its classification, (b) within-word segmental features, (c) age, and (d) speech disorder severity metrics for children with typical development (TD) or childhood apraxia of speech (CAS).

	Classification Accuracy for TD						Classification Accuracy for CAS					
	Single Pronunciation			Multiple Pronunciation			Single Pronunciation			Multiple Pronunciation		
**Confidence ^a^**												
Single pronunciation	0.73 **			—			0.39 **			—		
Multiple pronunciation	—			0.35 *			—			0.58 **		
**Segmental features ^a^**												
Nasal phoneme adjacent to vowel	−0.05			−0.13			−0.14			−0.12		
Liquid/glide adjacent to vowel	−0.28 *			−0.27			−0.21			−0.26		
Nonschwa unstressed vowel	−0.35 *			−0.33 *			−0.22			−0.25		
Long stressed vowel	0.01			0.02			−0.21			0.01		
Unvoiced plosive + schwa vowel	−0.09			−0.02			−0.20			−0.09		
	**Single Pronunciation**			**Multiple Pronunciation**			**Single Pronunciation**			**Multiple Pronunciation**		
	**SW + WS**	**SW**	**WS**	**SW + WS**	**SW**	**WS**	**SW + WS**	**SW**	**WS**	**SW + WS**	**SW**	**WS**
**Age ^b^**	0.18	0.21	0.34	0.47	0.33	0.47	0.41 *	0.22	0.56 **	0.43 *	0.31	0.52 **
**Speech disorder severity ^b^**												
PCC	0.04	−0.10	0.38	0.58 *	0.47	0.42	0.33	0.38	0.28	0.45 *	0.46 *	0.40 *
PVC	0.11	0.05	0.42	0.35	0.23	0.40	0.39 *	0.41 *	0.34	0.50 *	0.49 *	0.45 *
PPC	0.08	0.02	0.38	0.40	0.24	0.38	0.36	0.40 *	0.31	0.48 *	0.48 *	0.43 *

Note: ^a^ Spearman correlation coefficient; ^b^ Spearman correlation coefficient for TD children, Pearson correlation coefficient for CAS children used for comparison with confidence estimates, SW = strong–weak (e.g., dinosaur), WS = weak–strong (e.g., tomato); PCC = percent consonants correct, PVC = percent vowels correct, PPC = percent phonemes correct; * *p* < 0.05, ** *p* < 0.01 level (2-tailed).

## Data Availability

Data are available through the corresponding author. Some data may be subject to privacy constraints.

## Appendix A

Stimuli of the Gozzard Single-Word Test of Polysyllables (in alphabetical order)

Aeroplane

Ambulance

Animals

Avocado

Banana

Broccoli

Bulldozer

Butterfly

Capsicum

Caterpillar

Cauliflower

Computer

Crocodile

Cucumber

Dinosaur

Echidna

Elephant

Escalator

Hamburger

Helicopter

Hippopotamus

Hospital

Kangaroo

Koala

Medicine

Microwave

Mosquito

Motorbike

Octopus

Pinocchio

Platypus

Policeman

Potato

Pyjamas

Rectangle

Rhinoceros

Sausages

Spaghetti

Stethoscope

Television

Thermometer

Tomato

Triangle

Umbrella

Vacuum cleaner

Vegemite

Vegetables

Washing machine

Watermelon

Zucchini
